# Impact of *Hericium erinaceus* and *Ganoderma lucidum* metabolites on AhR activation in neuronal HT-22 cells

**DOI:** 10.1007/s43440-025-00767-w

**Published:** 2025-08-14

**Authors:** Anna Tabęcka-Łonczyńska, Bartosz Skóra, Dominika Szlachcikowska, Rafał Jastrząb, Małgorzata Anna Marć, Jennifer Mytych, Oliwia Koszła, Przemysław Sołek, Konrad A. Szychowski

**Affiliations:** 1 Department of Biotechnology and Cell Biology, Medical College, University of Information Technology and Management in Rzeszów, Sucharskiego 2, Rzeszów, 35-225 Poland; 2 Research and Development Center, Olimp Laboratories Sp. z o.o, Pustynia 84F, Dębica, 39-200 Poland; 3 Department of Biopharmacy, Medical University of Lublin, Chodźki 4a, Lublin, 20-093 Poland; 4 Department of Biochemistry and Toxicology, University of Life Sciences, Akademicka 13, Lublin, 20-950 Poland

**Keywords:** *Hericium erinaceus*, *Ganoderma lucidum*, HT-22 cell line, M-CFS extract, Cognitive functions

## Abstract

**Background:**

The proper functioning of the nervous system determines the homeostasis of the entire body. There are many known approaches designed to positively stimulate the functions of the central nervous system by applying various plants and fungal extracts, but their course of action is poorly understood. *Hericium erinaceus* and *Ganoderma lucidum* are examples of fungi with medicinal properties and with a positive health-promoting effect. Therefore, the aim of our study was to evaluate the effect of *H. erinaceus* or *G. lucidum* M-CFS with their active metabolites alone and/or in co-treatment with CAY10464 [antagonist of aryl hydrocarbon receptor (AhR)] on the metabolic parameters, cell cycle, and selected protein expression.

**Methods:**

The study was based on the use of the resazurin reduction assay, flow cytometry analyses, and Western blotting in the mouse hippocampal neuronal cell line (HT-22) in vitro.

**Results:**

The obtained results proved no cytotoxicity of the tested metabolites towards the HT-22 cells in the concentration range of 2.5% − 10% of culture medium. The cells treated with the tested compounds were characterized by an increase in the protein expression of SQSTM/p62, PCNA, c-SRC, SOD1, AhR, Beclin 1, and ERK1/2. Moreover, a significant role of AhR in the mechanism of action of the tested metabolites was observed at the protein expression level.

**Conclusion:**

The observed increase in the proliferation-related markers in the HT-22 cells proves the beneficial protective potential of these M-CFSs. Given the findings, we speculate their positive impact on the cognitive functions in the central nervous system.

**Clinical trial registration date:**

Not applicable.

**Clinical trial number:**

Not applicable.

**Graphical Abstract:**

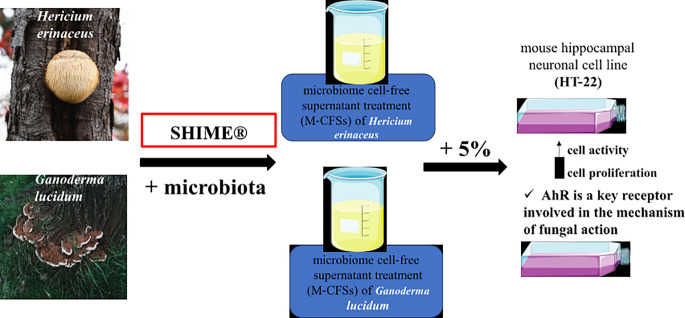

**Supplementary Information:**

The online version contains supplementary material available at 10.1007/s43440-025-00767-w.

## Highlights

5% microbiome cell-free supernatant with *H. erinaceus* or *G. lucidum* does not exert cytotoxicity towards the HT-22 cell line.

*H. erinaceus* and *G. lucidum* show a pro-proliferative effect on the HT-22 cell line.

Metabolites of M-CFS with *H. erinaceus* or *G. lucidum* influence the HT-22 cell line *via* the AhR pathway.

**Supplementary Information:**

The online version contains supplementary material available at 10.1007/s43440-025-00767-w.

## Introduction

Gut microbiota modulates the development and homeostasis of the central nervous system (CNS) [1] by complex bidirectional brain-gut-microbiome (BGM) interactions [2]. There is evidence that intestinal microbial metabolites modulate aryl hydrocarbon receptor (AhR) activity, which enables metabolic communication between the host and the gut microbiota [3].

AhR is an evolutionarily conserved transcription factor present in various types of cells, e.g., immune system cells [4], hepatic cells [5], glial cells [6, 7], and neuronal cells [8]. It constitutes an environmental sensor mediating the response to the toxic and biological effects of dioxins in the environment and endogenous as well as exogenous metabolites [9]. AhR is an important receptor in both physiological and pathological processes in the body. To date, many different dietary phytochemicals have been identified as potential ligands [10, 11]. It was found that flavonoids, one of the largest bioactive groups of food-originating compounds influence the regulation and activation of AhR [12, 13]. Also, products of amino acid metabolism [14, 15], especially tryptophan derivatives − 6-formylindolo[3,2-b]carbazole and kynurenine, are AhR ligands [16]. Both chemical groups were identified and present in *Hericium erinaceus* (*H. erinaceus*) and *Ganoderma lucidum* (*G. lucidum*) [17, 18]. Products of natural origin contain important ingredients that have been used for millennia in the pharmacotherapy of many health problems [15, 19]. Fungi as functional food are a rich source of bioactive compounds [20]. Moreover, fungal products containing metabolites are used as supporting supplements in the treatment of many diseases, as in the case of *H. erinaceus* and *G. lucidum*, which exert particularly valuable pharmacological properties [21, 22].

*H. erinaceus* (lion’s mane mushroom; Yamabushitake; monkey’s head mushroom) originates in East Asia and is used in many countries for culinary and medicinal purposes [23]. *H. erinaceus* has numerous health properties, e.g., antioxidant [24], anticancer and antimicrobial [25], anti-inflammatory [26], and antihyperglycemic and hypolipidemic [27] activity. To date, multiple classes of compounds in *H. erinaceus*, with beneficial potential, have been discovered (benzaldehydes, terpenoids, alkaloids). The most important are hericenones and erinacines. They were able to penetrate the blood-brain barrier (BBB), showcasing a new alternative in the treatment of neurological disorders [28, 29]. *G. lucidum* (Reishi; Lingzhi; Youngzhi; Linh chi), similar to *H. erinaceus*, is widely used due to the content of many bioactive substances, such as triterpenes, flavonoids, phenolic compounds, glycoproteins, proteoglycans, and polysaccharides [18, 30–32]. Moreover, numerous properties of bioactive substances contained in *G. lucidum* have been demonstrated so far, including antioxidant [33], hypoglycemic [34], immunomodulatory [35], antidiabetic [36, 37], and anticancer [38] activity. The neuroprotective effect of *G. lucidum* on brain injury and neurodegenerative diseases is also very promising [39]. Also, at least several studies on this fungus have been conducted, confirming its health-promoting effects on the brain. The influence of *G. lucidum* in the treatment of cognitive disorders associated with neurodegenerative diseases has been demonstrated [40], supporting the dissolution or removal of senile plaques and neurofibrillary tangles (NFTs) in the brain of a mouse model of Alzheimer’s disease and reducing amyloid angiopathy [41]. *G. lucidum* extracts prevented the production of pro-inflammatory and cytotoxic factors in dopaminergic neurons and microglia [42], and studies on primary cultures of dopaminergic cells from embryonic mice showed stimulation of regeneration of brain damage [43]. However, there is no literature data on the effect of the microbiome cell-free supernatant (M-CFS) of both fungal species on neuronal cell models or on their impact on the AhR receptor, whose role is crucial in the gut-brain communication pathway.

Taking the above into account, our study aimed to elucidate the molecular mechanisms of gut-brain signaling and to investigate whether biological factors synthesized during the digestion of *H. erinaceus* or *G. lucidum* fungi can support the functioning of the nervous system by affecting the AhR receptor. The study was carried out using the dynamic stimulator of the human intestinal microbial ecosystem (SHIME^®^) and mouse hippocampal cells (line HT-22) as a model of neuronal cells in vitro. The basic metabolic parameters (metabolic activity, cell cycle analysis, LDH release, caspase-3 activity) and the protein expression of sequestosome 1 (SQSTM/p62), proliferating cell nuclear antigen (PCNA), proto-oncogene tyrosine-kinase (c-SRC), superoxide dismutase-1 (SOD1), AhR, Beclin1, and extracellular signal-regulated kinas-1/2 (ERK1/2) were measured.

## Materials and methods

### Reagents

Dulbecco’s Modified Eagle Medium (DMEM) without phenol red (10–013-CVR) and phosphate buffer saline (PBS) without Ca^2+^ and Mg^2+^ were purchased from Corning (Corning, USA). Fetal bovine serum (FBS) and Perfect™ Tricolor Protein Ladder were purchased from EURx (Gdańsk, Poland). Trypsin, penicillin, streptomycin, ethanol, methanol, Tris-HCl, Tris-Base, Glycine, acrylamide/bisacrylamide (37:1, 2.6% crosslinker), sodium-dodecyl sulfate, ammonium persulfate, N,N, N′,N′-tetramethyl ethylenediamine (TEMED), resazurin sodium salt, lithium lactate, iodonitrotetrazolium chloride, β-nicotinamide adenine dinucleotide (NAD), propidium iodide (PI), protease inhibitor cocktail, bovine serum albumin (BSA), Bradford reagent, 1-methoxy-5-methylphenazinium methyl sulfate (MPMS), caspase-3 colorimetric substrate (Ac-DEVD-pNA), HEPES, sodium chloride (NaCl), 3-[(3-cholamidopropyl)dimethylammonio]-1-propanesulfonate hydrate (CHAPS), ethylenediaminetetraacetic acid (EDTA), glycerol and DL-dithiothreitol (DTT), iodonitrotetrazolium salt (INT), dipotassium phosphate (K_2_HPO_4_), monobasic potassium phosphate (KH_2_PO_4_), sodium thioglycolate (C_2_H_3_NaO_2_S), and sodium thionite (Na_2_S_2_O_4_) were purchased from Sigma-Aldrich (Saint Louis, USA). The RNAse, sodium hydroxide (NaOH), and hydrochloric acid (HCl) were purchased from VWR (Gdańsk, Poland). Specific antibodies against β-actin, SOD1, and a PVDF membrane with 0.45 μm pore size were purchased from Santa Cruz Biotechnology (Santa Cruz, USA). Specific antibodies against AhR were obtained from Proteintech GmbH (Düsseldorf, Germany). Specific antibodies against SQSTM/p62, PCNA, c-SRC, and ERK1/2 were purchased from ABClonal (Woburn, USA), and antibodies against Beclin 1 were provided by ThermoFisher (Waltham, USA). 1,3-dichloro-5-[(1E)-2-(4-methoxyphenyl)ethenyl]-benzene (CAY10464) was purchased from Cayman Chemicals (Ann Arbor, USA). Adult L-SHIME growth medium, pancreatic enzymes, bile salts, and sodium hydrogen carbonate (NaHCO_3_) were purchased from ProDigest BV (Gent, Belgium).

### Simulator of human microbial intestinal ecosystem (SHIME^®^) experiment

Following the research conducted by Koszła et al. [44], we evaluated the mutual reactions of the *H. erinaceus* or *G. lucidum* fungi with the human microbiome by experimenting in the stimulator of the human intestinal microbial ecosystem (SHIME^®^, ProDigest-Ghent University, Ghent, Belgium), as previously described (Van de Wiele et al., 2015). The M-CFS composition was previously characterized by Koszła et al. [44] using HPLC-ESI-QTOF-MS/MS.

In brief, the experimental setup included two reactors: one simulating the stomach and small intestine and the other simulating the proximal colon (PC, 500 mL, pH 5.6–5.9). These reactors were maintained in anaerobic conditions at a constant temperature of 37 °C, with stirring at 200 rpm. The colon compartment was inoculated with fresh fecal samples from a healthy 31-year-old male donor, following established protocols and ethical guidelines [45]. Over three weeks of stabilization and one week of control, fungal extracts were administered. Briefly, 500 mg of fungal extracts (*Ganoderma lucidum* and *Hericium erinaceus*) were prepared, suspended in 1 mL of PBS, thoroughly vortexed, and then administered into the appropriate proximal colon compartments of the SHIME^®^ model using a syringe. Three independent experimental setups were used: the first (control) received only PBS, the second received the *G. lucidum* extract, and the third received the *H. erinaceus* extract. This procedure was carried out once daily over three weeks. At the end of the experiment, final samples were collected, sterilized using a 0.2 μm filter, and stored as microbiome cell-free supernatant (M-CFS) for subsequent analysis.

### Cell culture

The mouse hippocampal neuronal cell line (HT-22, SCC129) was purchased from MERCK (Millipore). The cells were cultured in DMEM without phenol red, supplemented with 10% FBS and 0.1% penicillin/streptomycin at 37 °C with 5% CO_2_ until reaching 80% confluency. Afterwards, the cells were collected by trypsinization and seeded on 96-well plates (for resazurin reduction assay, LDH-release assay, and caspase-3 activity analysis), ⌀100 mm culture dishes (Western Blot), or T25 culture flasks (flow cytometry) at the density of 3.5 × 10^3^ cells/well, 9 × 10^5^ cells/dish, or 1 × 10^6^ cells/flask, respectively, 24 h before the experiment. Next, the medium was removed, and fresh medium containing 2.5%, 5%, and 10% of the microbiome cell-free supernatant (M-CFSs) of *H. erinaceus* or *G. lucidum* (dose-response analysis) was applied. These concentrations were chosen empirically as a representative of a high dose of fungi-derived active substances. For co-treatments, a 5% *v/v* M-CFS concentration was used, based on the intermediate efficacy observed in the dose–response trials, to allow detection of both potentiation and attenuation of M-CFS effects. Next, 5% of M-CFSs of *H. erinaceus* or *G. lucidum* alone or in co-treatment with 1 µM of CAY10464 (selective AhR antagonist) were added for a specified period (depending on methods). Before adding to the culture medium, M-CFS was filtered through a 0.22 μm-pore size filter to remove remaining bacteria from the suspension. The control group was always composed of cells treated with an equal volume of control M-CFS (without the mushrooms). The concentration of CAY10464 was chosen based on previous reports [46, 47].

### Resazurin reduction assay

The resazurin reduction assay was performed as described by Skóra et al. [48] without modifications. After specified periods (24 h and 48 h), the fluorescence measurement was performed at an excitation wavelength of 570 nm and an emission wavelength of 590 nm using a microplate reader (FilterMax F5). The results were expressed as percentages (%) of the control.

### Lactate dehydrogenase (LDH) release assay

The LDH release assay was performed as described by Kaja et al. [49] with modifications. After 24 h and 48 h, 80 µL of the post-culture medium was transferred to a new 96-well plate. Subsequently, 50 µL of the reaction mixture (4 mM INT, 6.4 mM NAD, 320 mM lithium lactate, 150 mM MPMS in 220 mM Tris-HCl, pH = 8.2) was added to each well and incubated in the dark at room temperature for 30 min. After this time, the absorbance was measured at the 450 nm wavelength using a microplate reader (FilterMax F5). The results were expressed as percentages (%) of the control. The cells were subsequently used in the caspase-3 activity assay.

### Caspase-3 activity assay

The caspase-3 activity assay was carried out as described previously without modifications [50]. After the treatment of the cells with the specific compounds for 24 h and 48 h, the caspase-3 colorimetric substrate (Ac-DEVD-pNA) and the absorbance were measured at the 405 nm wavelength after 30 min. using a microplate reader (FilterMax F5). The results were expressed as a percentage (%) of the control.

### Cell cycle analysis

After the 24-h treatment of the HT-22 cells with the medium containing 5% of *H. erinaceus* or *G. lucidum* M-CFS, the cells were collected by trypsinization. The cells were washed once with PBS and centrifuged for 6 min., 600 × g. After this step, the cells were fixed with ice-cold 70% ethanol for 20 min. at -20 °C. Subsequently, the pellets were washed once in cold PBS, followed by centrifugation (5 min., 600 ×g), and stained with a mixture containing 50 µg/mL of propidium iodide (PI) and 1 mg/mL of RNAse (in PBS) for 30 min. at RT. Next, the cell suspensions were subjected to cell cycle analysis using a flow cytometer (BD Accuri C6 Plus) equipped with an autosampler (CSampler Plus). Next, the fluorescence of PI was assessed using the FL-2 channel. A total of 100,000 events were acquired.

### Western blot

The Western Blot method was performed as described in a previous study without modifications [48]. The protein concentration was determined by the bicinchoninic acid (BCA) quantitation assay using BSA as a standard. Next, the concentrations in all samples were standardized. Forty micrograms of protein were loaded on an SDS gel. The β-actin protein expression was used as a loading control for each sample after stripping the membrane post-detection with buffer containing 1.5% glycine, 0.1% SDS, and 1% Tween-20 (pH = 2.2). The antibodies, their dilutions, and catalog numbers/producers are listed in the table below (Table 1). The band intensity was measured using GelQuantNET free software (http://biochemlabsolutions.com/GelQuantNET) normalized to β-actin, and compared to the control.

**Table 1:** Primary and secondary antibodies (HRP-conjugated) with target protein, host species, dilution, producers, and catalog numbers used in the Western blot method

Primary antibody			Secondary antibody (HRP-conjugated)		
Antibody target (host species)	Dilution	Producer (cat. number)	Antibody target (host species)	Dilution	Producer (cat. number)
anti-SQSTM/p62 (Rb)	1:2000	ABClonal (A11250)	Anti-Rb-HRP-conjugated (Gt)	1:10000	ThermoFisher (31460)
anti-PCNA (Rb)	1:2000	ABClonal (A12427)			
anti-c-SRC (Rb)	1:4000	ABClonal (A19119)			
anti-ERK1/2 (Rb)	1:2000	ABClonal (A16686)			
anti-Beclin 1 (Rb)	1:2000	ThermoFisher (PA1-16857)			
anti-AhR (Ms)	1:3000	Proteintech GmbH (67785-1-Ig)	Anti-Ms-HRP-conjugated (Gt)	1:5000	ThermoFisher (31430)
anti-SOD1 (Ms)	1:400	Santa Cruz Bt. (sc-101523)			
anti-β-actin (Ms)	1:1500	Santa Cruz Bt. (sc-47778)		1:10000	

Ms – mouse; Rb – rabbit; Gt – goat, HRP – horseradish peroxidase

### Statistical analysis

The data presented in the graphs are means with SD (standard deviations) of at least three independent experiments (*n* ≥ 3), while the description of the results presents the F_df_ and p values. These data were subsequently used in the statistical analysis, which was performed using one-way ANOVA or two-way ANOVA (for cell cycle-based analysis) in GraphPad Prism 8.0 Statistical Panel with Tukey’s *post-hoc* test (for one-way as well as two-way ANOVA). Data in graphs denoted as *, **, and *** are statistically different at *p* < 0.05, *p* < 0.01, and *p* < 0.001, respectively, vs. the control (cells treated with the growth medium containing an equal volume of control M-CFS), while means marked as # differs statistically at *p* < 0.05 between each other (Tukey’s *post-hoc* test).

## Results

### Metabolic activity and cell cycle analysis

**Fig. 1:**
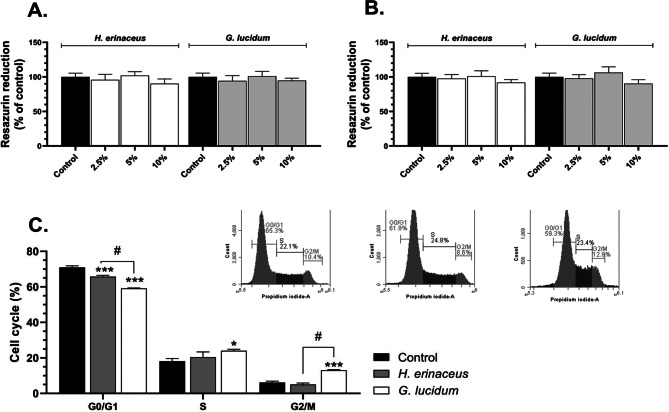
Metabolic activity measured by resazurin reduction method (**A** – **B**) of HT-22 cells treated with the growth medium containing 2.5% − 10% of the microbiome cell-free supernatant (M-CFS) of *H. erinaceus* and *G. lucidum* for 24 h (**A**) and 48 h (**B**). Cell cycle flow cytometry analysis, using propidium iodide staining (**C**) of HT-22 cells treated with the medium containing 5% of the control M-CFS, the M-CFS of *H. erinaceus* or *G. lucidum*. The representative cytograms show the gating strategy (insets). The bars in the graphs represent mean values with SD of at least 3 independent experiments (*n ≥ 3*). Data marked as * and *** are statistically different at *p* < 0.05 and *p* < 0.001, respectively, compared to the control (one-way ANOVA; Tukey’s *post-hoc* test or two-way ANOVA for cell cycle-based analysis; Tukey’s *post-hoc* test), while means denoted as # were used to determine the statistical difference between these two data at *p* < 0.05 (one way ANOVA; Tukey’s *post-hoc* test or two-way ANOVA for cell cycle-based analysis; Tukey’s *post-hoc* test)

The cells treated with *H. erinaceus* M-CFS did not affect the metabolic activity in any tested concentration, after either 24 h (F_3,20_=1.518, *p* = 0.2406, one-way ANOVA followed by Tukey’s test) or 48 h (F_3,20_=2.119, *p* = 0.1298, one-way ANOVA followed by Tukey’s test) (Fig. 1A, B). Similarly, the HT-22 cells treated with growth medium containing 2.5%, 5%, or 10% of *G. lucidum* M-CFS exhibited no significant changes in the metabolic activity after 24 h (F_3,20_=1.997, *p* = 0.1468, one-way ANOVA followed by Tukey’s test) and 48 h (F_3,20_=1.906, *p* = 0.1611, one-way ANOVA followed by Tukey’s test) (Fig. 1A, B).

The effects of treatment (F₂,₁₈ = 4.585, *p* = 0.0246), cell cycle phase (F₂,₁₈ = 5316, *p* < 0.001), and their interaction (F₄,₁₈ = 59.05, *p* < 0.001) in the cell cycle-based experiments were statistically significant (two-way ANOVA followed by Tukey’s test). Treatment with *H. erinaceus* M-CFS caused a significant decrease (*p* = 0.0002) in the cell population in the G0/G1 phase compared to the control, while no significant changes were observed in the S (*p* = 0.0920) or G2/M (*p* = 0.5290) phases (Fig. 1C). Conversely, HT-22 cells treated with *G. lucidum* M-CFS showed a significant decrease (*p* < 0.001) in the G0/G1 cell population compared to the control. Additionally, there was a significant increase (*p* < 0.001) in the populations of cells in both the S phase and the G2/M phase following *G. lucidum* M-CFS treatment (Fig. 1C).

### Toxicity and proapoptotic activity

**Fig. 2:**
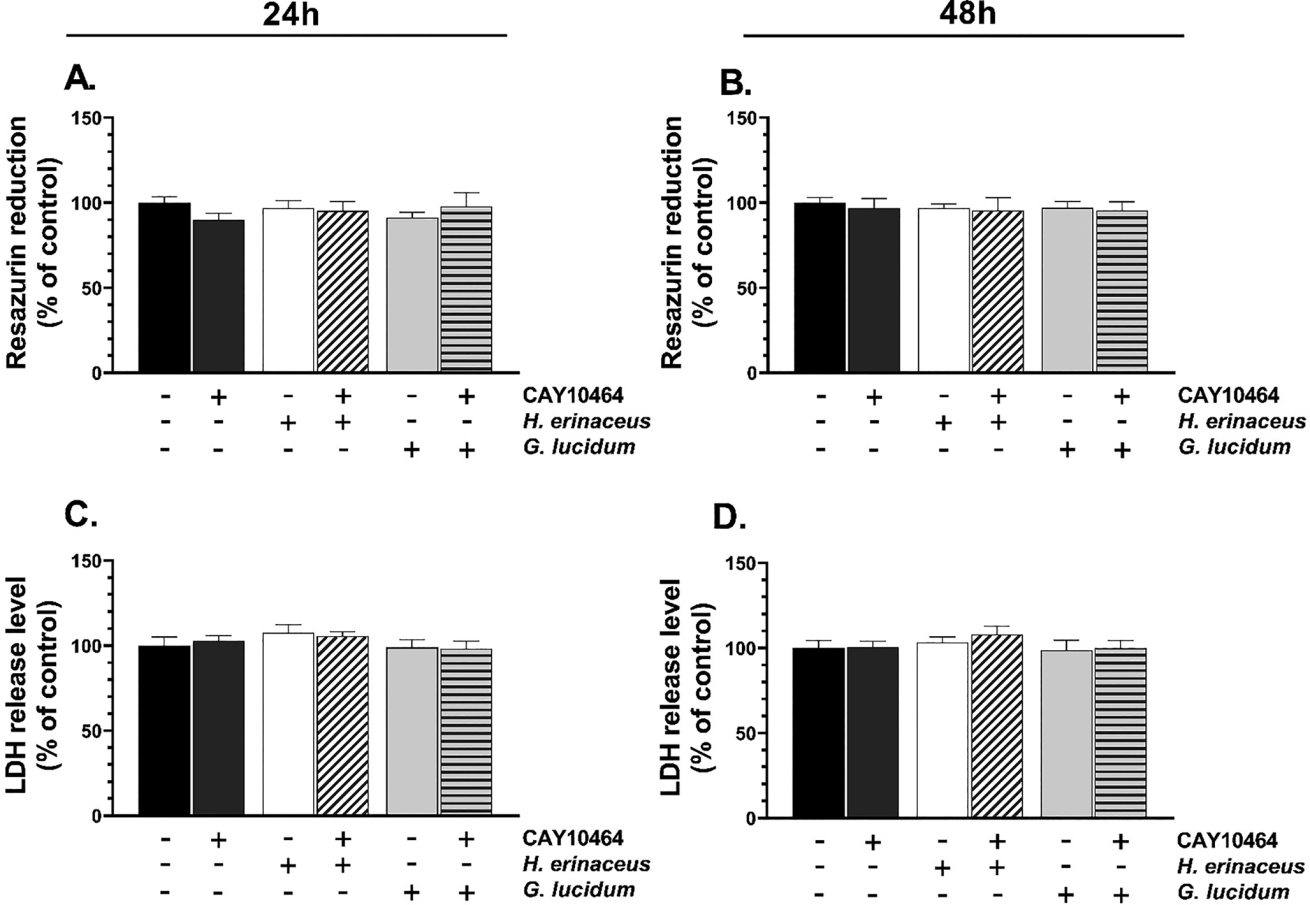
Metabolic activity – measured by resazurin reduction method (**A**, **B**), and lactate dehydrogenase (LDH) release level (**C**, **D**) of HT-22 cells treated with the growth medium containing 5% of the microbiome cell-free supernatant (M-CFS) of *H. erinaceus* or *G. lucidum* and 1 µM CAY10464 alone or in co-treatment for 24 h (**A**, **C**) and 48 h (**B**, **D**). The bars in the graphs represent means with SD of at least 3 independent experiments (*n ≥ 3*); (one-way ANOVA; Tukey’s *post-hoc* test)

The HT-22 cells treated with *H. erinaceus* or *G. lucidum* M-CFS and CAY10464 alone or in co-treatment did not show any significant changes in the metabolic activity after 24 h (F_5,30_=0.9842, *p* = 0.4436, one-way ANOVA followed by Tukey’s test) and 48 h (F_5,30_=0.7167, *p* = 0.6159, one-way ANOVA followed by Tukey’s test) (Fig. 2A, B). Similarly, the LDH release assay did not show any significant changes in this parameter in any tested group after 24 h (F_5,30_=1.479, *p* = 0.2259, one-way ANOVA followed by Tukey’s test) and 48 h (F_5,30_=0.9664, *p* = 0.4539, one-way ANOVA followed by Tukey’s test) (Fig. 2C, D).

**Fig. 3:**
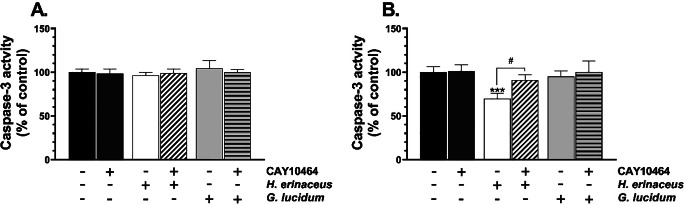
Caspase-3 activity level was measured using colorimetric assay in HT-22 cells treated with the microbiome cell-free supernatant (M-CFS) of *H. erinaceus* or *G. lucidum* and 1 µM CAY10464 alone or in co-treatment for 24 h (**A**) and 48 h (**B**). The bars in the graphs represent means with SD of at least 3 independent experiments (*n ≥ 3*); (one-way ANOVA; Tukey’s *post-hoc* test). Data denoted as *** are statistically different at *p* < 0.001 (one-way ANOVA; Tukey’s *post-hoc* test), compared to the control, while data denoted as # are statistically different between each other at *p* < 0.05 (one-way ANOVA; Tukey’s *post-hoc* test)

The caspase-3 activity level after the 24-h treatment of the cells with any tested extract did not change significantly (F_5,30_=1.725, *p* = 0.1594, one-way ANOVA followed by Tukey’s test) (Fig. 3A). On the other hand, the cells treated with tested compounds for 48 h significantly affect this parameter (F_5,30_=13.93, *p* < 0.001, one-way ANOVA followed by Tukey’s test) (Fig. 3B). Specifically, the *H. erinaceus* M-CFS alone caused a significant decrease in the caspase-3 activity (*p* = 0.00003), compared to the control (Fig. 3B). In turn, the caspase-3 activity level increased (*p* = 0.00008) in HT-22 treated with *H. erinaceus* M-CFS and co-treated with CAY10464, compared to cells treated with *H. erinaceus* M-CFS alone (Fig. 3B).

### Protein expression

**Fig. 4:**
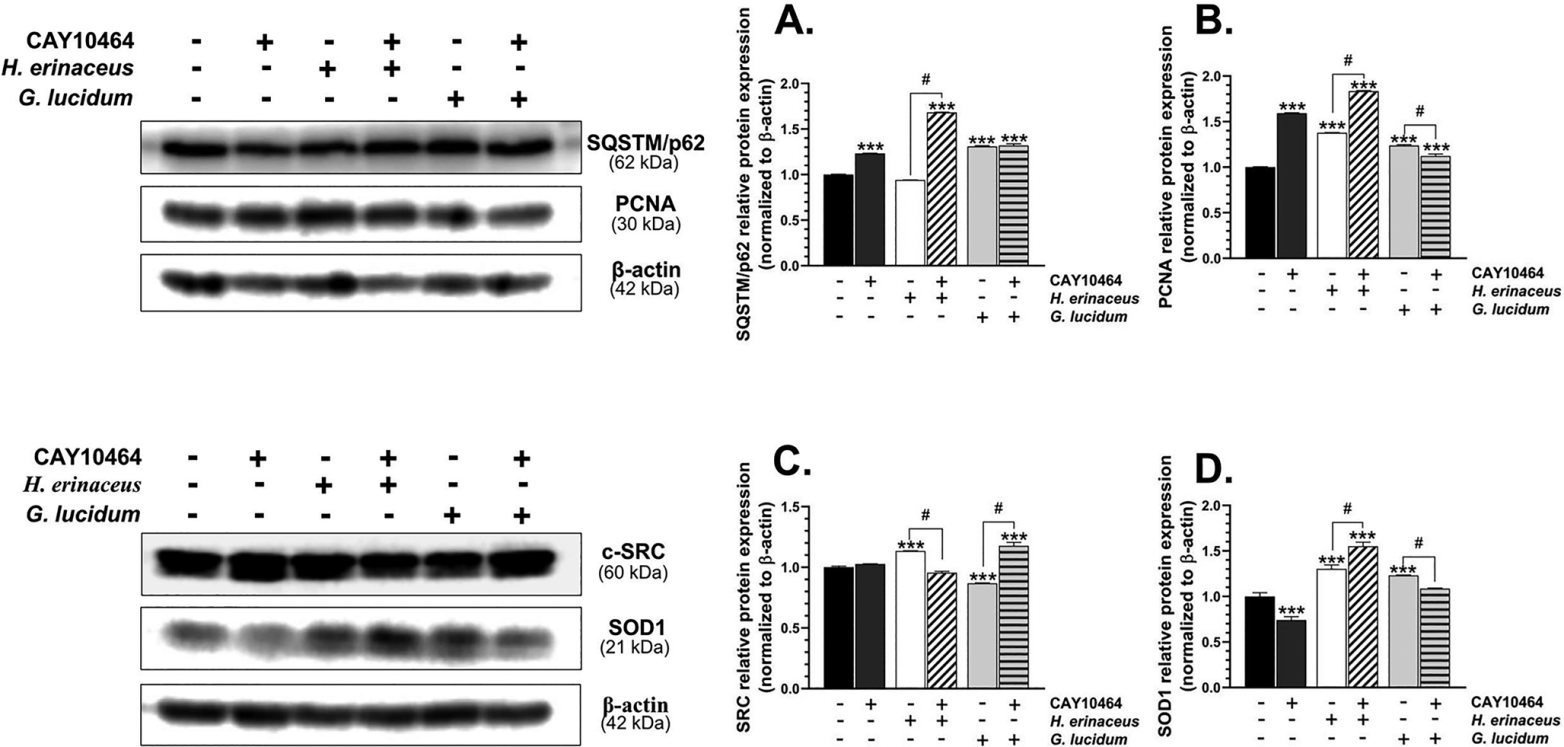
Protein expression of SQSTM/p62 (**A**), PCNA (**B**), c-SRC (**C**), and SOD1 (**D**) was determined using Western blot analysis in HT-22 cells treated with the microbiome cell-free supernatant (M-CFS) of *H. erinaceus* or *G. lucidum*, 1 µM CAY10464 alone, and co-treatment of the cells for 24 h. The bars in the graphs represent means with SD of at least 3 independent experiments (*n ≥ 3*). Data denoted as *** are statistically different at *p* < 0.001 (one-way ANOVA; Tukey’s *post-hoc* test), compared to the control, while data denoted as # are statistically different from each other at *p* < 0.05 (one-way ANOVA; Tukey’s *post-hoc* test). The representative blots are shown on the left

The treatment with the tested compounds significantly affects the expression of the SQSTM1/p62 protein (F_5,12_=2760.94, *p* < 0.001, one-way ANOVA followed by Tukey’s test). This protein expression increased in the cells treated with CAY10464 (*p* < 0.001), *H. erinaceus* M-CFS/CAY10464 (*p* = 0.000028), *G. lucidum* (*p* < 0.001), and *G. lucidum* M-CFS/CAY10464 (*p* < 0.001), compared to the control (Fig. 4A). Moreover, the HT-22 cells treated with *H. erinaceus* M-CFS/CAY10464 were characterized by an increase in the expression of this protein (*p* < 0.001), compared to cells treated with *H. erinaceus* M-CFS alone (Fig. 4A).

The expression of PCNA protein changed significantly after treatment (F_5,12_=2517, *p* < 0.001, one-way ANOVA followed by Tukey’s test). The cells in all the analyzed groups were characterized by an increase in the PCNA protein expression (for all samples *p* < 0.001), compared to the control (Fig. 4B). In turn, a significant increase (*p* < 0.001) in the expression of this protein was observed in HT-22 treated with *H. erinaceus* M-CFS/CAY10464, compared to cells treated with *H. erinaceus* M-CFS alone (Fig. 4B). Similarly, the *G. lucidum* M-CFS/CAY10464 treatment caused a significant decrease (*p* < 0.001) in the PCNA protein expression, compared to the cells treated with *G. lucidum* M-CFS (Fig. 4B).

The tested compounds significantly affect c-SRC protein expression (F_5,12_=215.6, *p* < 0.001, one-way ANOVA followed by Tukey’s test). However, a significant increase was observed only in the cells treated with *H. erinaceus* M-CFS (*p* < 0.001) and *G. lucidum* M-CFS/CAY10464 (*p* < 0.001), compared to the control (Fig. 4C). On the other hand, it decreased in the cells treated with *G. lucidum* M-CFS (*p* < 0.001), compared to the control (Fig. 4C). In turn, a decrease in the expression of this protein in the HT-22 cells treated with *H. erinaceus* M-CFS/CAY10464 (*p* < 0.001) was observed, compared to the cells treated with *H. erinaceus* M-CFS alone (Fig. 4C). Conversely, an increase in the expression (*p* < 0.001) was observed in the *G. lucidum* M-CFS/CAY10464-co-treated cells, compared to HT-22 treated with *G. lucidum* M-CFS alone (Fig. 4C).

The expression of the SOD1 protein changed significantly after treatment with the tested compounds (F_5,12_=200.3, *p* < 0.001, one-way ANOVA followed by Tukey’s test). An increase in this parameter was observed in the cells treated with *H. erinaceus* M-CFS (*p* < 0.001), *H. erinaceus*/CAY10464 (*p* < 0.001), and *G. lucidum* M-CFS (*p* < 0.001), compared to the control (Fig. 4D). On the other hand, the SOD1 protein expression decreased (*p* < 0.001) in the cells treated with CAY10464 (Fig. 4D). Moreover, an increase in the expression of this protein was observed in the cells co-treated with *H. erinaceus* M-CFS/CAY10464 (*p* < 0.001), compared to HT-22 treated with *H. erinaceus* M-CFS alone (Fig. 4D). Furthermore, the SOD1 expression decreased (*p* < 0.001) in the *G. lucidum* M-CFS/CAY10464 variant, compared to the cells treated with *G. lucidum* M-CFS alone (Fig. 4D).

**Fig. 5:**
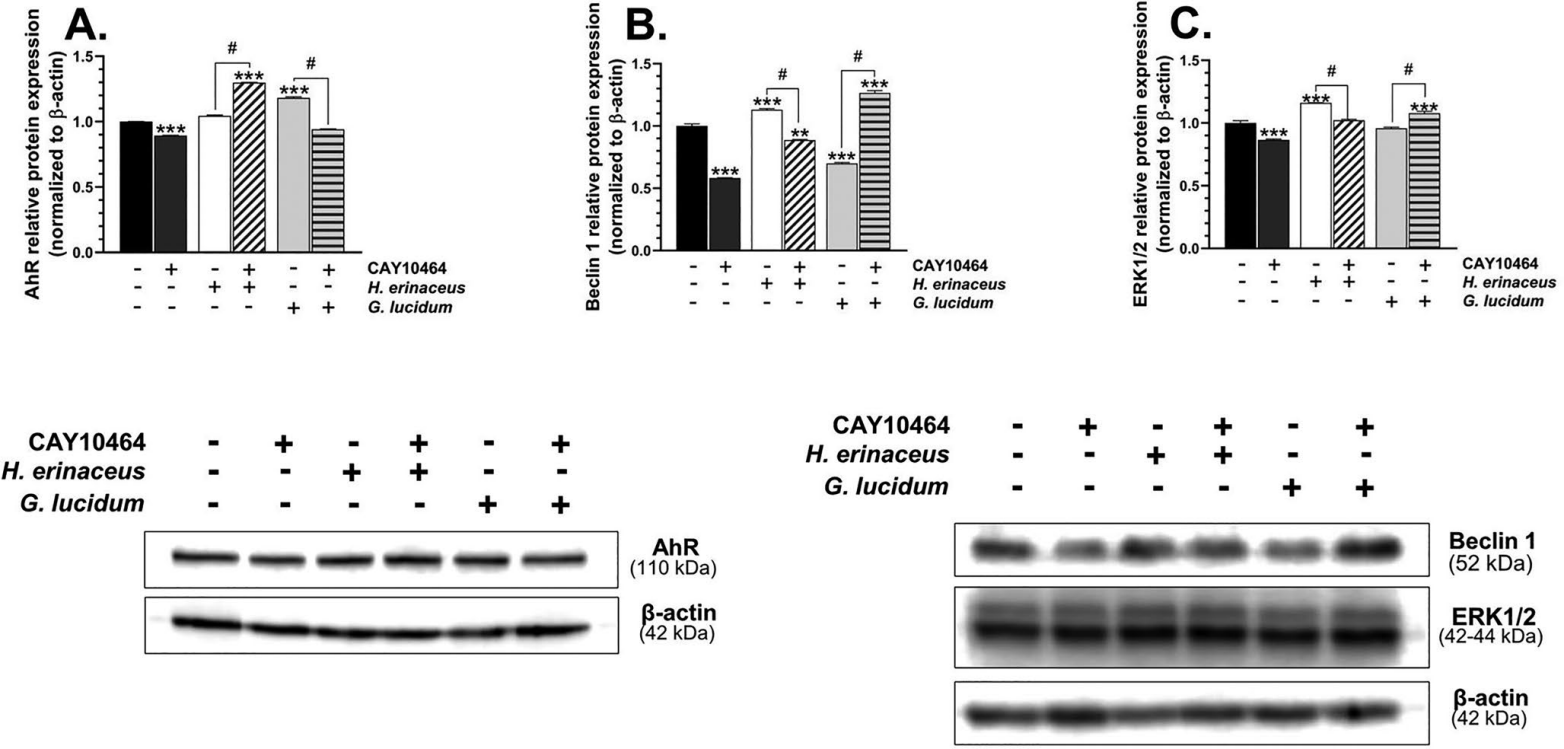
Protein expression of AhR (**A**), Beclin1 (**B**), and ERK1/2 (**C**) was determined using Western blot analysis in HT-22 cells treated with the microbiome cell-free supernatant (M-CFS) of *H. erinaceus*, *G. lucidum*, 1 µM CAY10464 alone, and co-treatment of the cells for 24 h. The bars in the graphs represent means with SD of at least 3 independent experiments (*n ≥ 3*). Means denoted as ** or *** are statistically different at *p* < 0.01 or *p* < 0.001 (one-way ANOVA; Tukey’s *post-hoc* test), compared to the control, while data denoted as # are statistically different from each other at *p* < 0.05 (one-way ANOVA; Tukey’s *post-hoc* test). The representative blots are shown in the figure

The treatment significantly affected AhR protein expression (F_5,12_=3314, *p* < 0.001, one-way ANOVA followed by Tukey’s test). A significant decrease (*p* < 0.001) in this protein expression was observed in cells treated with CAY10464 (Fig. 4A). On the other hand, an increase in the expression of this protein was observed in HT-22 co-treated with *H. erinaceus* M-CFS/CAY10464 (*p* < 0.001) and *G. lucidum* M-CFS alone (*p* < 0.001), respectively, compared to the control (Fig. 5A). In turn, an increase (*p* < 0.001) in the AhR protein expression was observed in the cells co-treated with *H. erinaceus* M-CFS/CAY10464, compared to HT-22 treated with *H. erinaceus* M-CFS alone (Fig. 5A). Conversely, the cells co-treated with *G. lucidum* M-CFS/CAY10464 were characterized by a decrease (*p* < 0.001) in the expression of this protein, compared to the cells treated with *G. lucidum* M-CFS alone (Fig. 5A).

Beclin 1 protein expression changed significantly in HT-22 cells after treatment with the tested compounds (F_5,12_=1583, *p* < 0.001, one-way ANOVA followed by Tukey’s test). CAY10464 significantly decreased (*p* < 0.001) the expression of this protein, while an increase in Beclin 1 was observed in cells treated with *H. erinaceus* M-CFS/CAY10464 (*p* < 0.001) and *G. lucidum* M-CFS alone (*p* < 0.001)), compared to the control (Fig. 5B). Additionally, the increase in the expression of this protein was detected in HT-22 treated with *H. erinaceus* M-CFS alone CAY10464 (*p* < 0.001) and *G. lucidum* M-CFS/CAY10464 (*p* < 0.001), compared to the control (Fig. 5B). Furthermore, a decrease (*p* < 0.001) in the Beclin 1 protein expression was observed in the cells treated with *H. erinaceus* M-CFS*/*CAY10464, compared to the cells treated with *H. erinaceus* M-CFS alone (Fig. 5B). In turn, an increase (*p* < 0.001) in the expression of this protein was shown in the cells treated with *G. lucidum* M-CFS/CAY10464, compared to HT-22 treated with *G. lucidum* M-CFS alone (Fig. 5B).

Treatment with the tested compounds significantly affected ERK1/2 protein expression (F₅,₁₂ = 237.1, *p* < 0.001; one-way ANOVA followed by Tukey’s test). The significant decrease in this parameter (*p* < 0.001) was observed only in the cells treated with CAY10464, compared to the control (Fig. 5C). On the other hand, an increase in the expression of this protein was observed in HT-22 treated with *H. erinaceus* M-CFS (*p* < 0.001) and *G. lucidum* M-CFS/CAY10464 (*p* < 0.001), compared to the control (Fig. 5C). Moreover, a decrease (*p* < 0.001) in the ERK1/2 protein expression was observed in the cells co-treated with *H. erinaceus* M-CFS and CAY10464, compared to the cells treated with *H. erinaceus* M-CFS alone (Fig. 5C). Conversely, an increase (*p* < 0.001) in the expression of this protein was observed in the cells co-treated with *G. lucidum* M-CFS/CAY10464, compared to HT-22 treated with *G. lucidum* M-CFS alone (Fig. 5C).

## Discussion

Our study is the first to examine the impact of *H. erinaceus* and *G. lucidum* M-CFS on the AhR-related effect in the HT-22 cell line. According to the literature, AhR is a conserved ligand-activated transcription factor sensitive to various environmental ligands, including dietary and microbial ones [51]. It has previously been shown that natural modulators may be compounds derived from food, plant microbiota metabolites, and endogenous tryptophan metabolites [52]. Gut microbiota is involved in the synthesis of biological metabolites in the digestive tract [53]. Among the identified metabolites, there are flavonoids from food [54] and microbiome metabolites like indoles [55–57]. It has been shown that they are physiological ligands for AhR, which, when activated, constitute a communication strategy between the digestive system and the brain [58, 59]. The intestinal microflora and the CNS are connected in two directions: through neuronal pathways, gastrointestinal metabolites, and immune signaling [60]. Such signaling is involved in maintaining intestinal homeostasis and affects the functioning of the CNS [61]. Studies conducted on flavonoids have shown that they may act as agonists and/or antagonists towards the activation of AhR transcription, thus constituting selective AhR modulators [62]. Among the neuroactive metabolites originating from microorganisms, an important role may be played by precursors or metabolites of tryptophan, which enter the circulatory system and reach the CNS [63].

Our experiment has shown that M-CFS with *H. erinaceus* and *G. lucidum* metabolites are not cytotoxic to mouse hippocampal neurons (HT-22 cell line). We did not find any significant changes in the metabolic activity in the tested cell line in both tested periods. These results are in line with data presented by Kushari et al. [21] and Sillapachaiyaporn et al. [64], who proved the protective effect of extracts from *H. erinaceus* or *G. lucidum* in the same cell model. Since there was no effect of the specific concentrations of any M-CFS, we chose 5% of the supernatants as a medium non-lethal concentration for the further part of the study. In turn, many papers have shown that AhR is a pivotal receptor that regulates the gut-brain axis due to its ability to be activated *inter alia* in resident cells in the CNS [61]. Therefore, in the next part of our study, we applied the co-treatment of the cells with the specific M-CFS and a selective AhR antagonist (CAY10464). However, no significant changes in the LDH release level were observed in the cells co-treated with *H. erinaceus*/CAY10464 or *G. lucidum*/CAY10464. However, after 24 h, the HT-22 cell cycle analysis showed an increase in the G2/M cell population in the *G. lucidum* M-CFS treatment, which may prove that the tested supernatants can be considered as safe in this cell model. After the 48-h exposure of the HT-22 cells to the M-CFS metabolites with *H. erinaceus*, we showed a significant decrease in caspase-3 activity. However, it is worth considering that caspase-3 may be involved in non-apoptotic processes, particularly in neuronal cells, where it supports mechanisms of synaptic plasticity and neural regeneration, as well as contributes to the removal of damaged cellular components without inducing cell death [65].

On the other hand, research conducted *inter alia* by Sun et al. (2017) [66] confirmed the neuroprotective effects of *G. lucidum* on rat cerebellar granule cells (CGCs), which was caused by the presence of specific polysaccharides in the tested *G. lucidum* extracts and their ability to significantly inhibit the apoptosis (proved by a decrease in the expression of caspase-3, Bax, Bim, and increased expression of Bcl-2) [67]. Moreover, Zhang et al. [68] demonstrated the ability of triterpenes isolated from *G. lucidum* to activate neurotrophin receptors, which consequently initiated neuronal survival pathways in a mouse fibroblast cell line (NIH-3T3). Besides, terpenes isolated from *G. lucidum* were able to significantly reduce the LPS-induced production of nitric oxide (NO) and the synthesis of pro-inflammatory cytokines in a murine macrophage cell line (RAW 264.7), which may indicate the neuroprotective effect of *G. lucidum* extract also in other cell types [69]. The anti-inflammatory effect of polysaccharides was also confirmed in mouse LPS-stimulated microglial cells, where inhibition of the production of NO and tumor necrosis factor (TNF-α) and the gene expression of inducible nitric oxide synthase (iNOS), cyclooxygenase (COX-2), and interleukin (IL)-1b was observed [61]. Moreover, oral administration of aqueous extract of *G. lucidum* influenced the stabilization of inflammatory markers in the rat hypobaric hypoxia test model [69].

Our study is the first to examine the impact of *H. erinaceus* and *G. lucidum* M-CFS on the AhR-related effects in the HT-22 cell line. Our results confirmed that the AhR is in some way involved in the mechanism of their action. We observed that, after the treatment of the cells with *H. erinaceus* alone, the AhR expression remained unchanged compared with the control. An increase in the AhR protein expression was observed following the co-treatment with *H. erinaceus* and CAY10464. While the mechanism of this change remains unclear, it may reflect an effect of the treatment on AhR protein stability or turnover. However, in the *G. lucidum* alone variant, the AhR expression increased, but no effect was observed with CAY10464. Therefore, we observed that both extracts engaged AhR in different ways. This is consistent with the results shown earlier, and AhR ligands derived from food flavonoids cause different effects depending on the exposure time and the species and tissue on which they act [70, 71]. These two medicinal mushrooms are known to contain polyphenolic compounds, including flavonoids such as quercetin, kaempferol, and related phenolics [15]. Notably, such dietary flavonoids can act as ligands for the aryl hydrocarbon receptor and modulate its activity [72, 73]. This raises the possibility that mushroom-derived flavonoids contributed to the AhR activation in our model. However, reduced levels of AhR agonists derived from the intestinal microflora have been demonstrated in many diseases [74, 75]. Interestingly, locomotor defects and changes in myelin structure were observed in AhR knockout studies in mice [76]. AhR deficiencies also lead to demyelination processes and the development of inflammation [77].

It has been reported that AhR may regulate the activity of certain kinases, *inter alia*, c-SRC, ERK1/2, and AKT [78, 79]. The results obtained in this study showed that the expression of the tested proteins is dependent on AhR participation. In the study conducted by Koszła et al. [44], M-CFS upregulated BDNF/CREB signaling in HT-22 cells, suggesting activation of pro-survival pathways, such as ERK1/2. Similarly, we observed increased ERK1/2 expression after the treatment with the M-CFS of *H. erinaceus* and with *G. lucidum*/CAY10464. Notably, the co-treatment with *H. erinaceus* M-CFS and CAY10464 reduced ERK1/2 levels, compared to M-CFS alone, indicating possible AhR-related modulation. These findings partially align with those reported by Koszła et al. [44] and suggest that ERK1/2 may be a key point of interaction between fungal metabolites and AhR signaling. Moreover, AhR is known to mediate gut-brain signals and thus exert an impact on inflammatory and neurodegenerative processes [80]. Based on the previous research, it is known that AhR controls cell cycle progression and differentiation, but this dependence is cell- and type-specific [81]. Our results in the HT-22 cell line exposed to the tested M-CFS seem to indicate this, although further studies are needed to confirm this issue. However, literature reports have shown that PCNA is increased in AhR-activated cells [82]. Interestingly, our studies showed an increase in the PCNA expression after the treatment with both extracts alone and with CAY10464, which probably helps maintain cell viability in a favorable environment through both AhR and another previously unexplored metabolic pathway. Although neurons are postmitotic, recent studies suggest that increased PCNA expression reflects activation of DNA repair and protective responses rather than cell proliferation. PCNA plays key roles in maintaining genome stability, coordinating oxidative stress responses, and facilitating DNA repair in neurons, particularly in the context of neurodegenerative diseases and injury [83]. Similarly, Ratto et al. [84] confirmed cell proliferation evidenced by an increase in the expression of PCNA in the hippocampus of mice treated with *H. erinaceus*.

AhR controls the expression of the SOD1 gene [85] and plays a role in neuronal signaling [86]. In human fetal pulmonary microvascular endothelial cells (HPMEC) with AhR deficit, a decrease in AhR expression, an increase in reactive oxygen species (ROS) formation, and a weakening of SOD1 activity were observed [86]. In our study, a significant increase in the SOD1 protein expression in the HT-22 cell line after the *H. erinaceus* M-CFS treatment and an even higher increase in the *H. erinaceus* with the CAY10464 variant were demonstrated. However, the expression increased only after the *G. lucidum* M-CFS alone treatment, whereas the expression was reduced in the *G. lucidum* with the CAY10464 variant. Although we did not measure the ROS level directly, the above-described changes in SOD1 may suggest that the M-CFSs of both mushrooms have antioxidant properties in the HT-22 cell line. Considering that, our previous analyses of the composition of the tested mushrooms [44] revealed that triterpenoids known to interact with AhR - such as ganoderic acids - are present exclusively in the *G. lucidum* extract. This likely accounts for the observed AhR-related activity and the effect of the receptor antagonist in the ex vivo experiments. In contrast, the *H. erinaceus* extract, which is rich in neurotrophic compounds such as hericenones and erinacines, does not exhibit comparable AhR activity. While we previously suggested potential antioxidant properties of the mushroom M-CFSs, direct evidence supporting this effect is lacking in the present study and requires further investigation. Overall, the distinct chemical profiles of the two extracts support the observed differences in AhR activity and reinforce the interpretation of the results obtained with the AhR antagonist. Additionally, the neuroprotective and anti-inflammatory effect of *H. erinaceus* mycelium was confirmed in other studies. After ischemic brain injury, the oxidative stress pathway in the endoplasmic reticulum was reduced by inactivation of mitogen-activated protein kinases p38/MAPK and a decrease in the expression of the enhancer-binding proteins (C/EBP) and homologous protein (CHOP), leading to a reduction of inducible iNOS and nitrotyrosine production [87]. Additionally, experiments with the use of *H. erinaceus* in the diet of mice (BALB/C) have shown anti-inflammatory effects, where an increase in glutathione (GSH) levels and amelioration of hepatic stress were observed in their plasma [88]. Additionally, research carried out on people aged 50–80 showed that *H. erinaceus* supplementation significantly increased cognitive function parameters, and no adverse effect was observed [89]. In vivo studies demonstrated the neuroprotective effect of *H. erinaceus* through a lipoxin A4 increase and an impact on stress *via* responsive vitagene proteins (intracellular redox system involved in neuroprotection) [90].

Basic autophagy is a physiological process important for maintaining homeostasis in nerve and brain cells [91]. Disturbances in this process result in neurodegenerative processes [92]. It has been shown that reduced levels of Beclin 1 are involved in the pathogenesis of Alzheimer’s disease [93]. The high expression of Beclin 1 and ERK1/2 proteins in our study, after the use of both extracts, may suggest the involvement of autophagy and activation of the survival and proliferation of hippocampal neuron cells. To date, numerous studies on mouse models with AhR gene knockout prove the importance of the AhR function both in maintaining cellular homeostasis and in pathophysiology [94–96]. Moreover, it is known that some endogenous AhR agonists formed as a result of tryptophan metabolism (kynurenine and kynurenic acid) can protect cells against apoptosis induced by oxidative stress in an AhR-dependent manner [97]. Lin and co-authors [98] used alcohol extracts of *H. erinaceus* in their research, which was administered to A53T transgenic mice with the expression of human α-synuclein as a research model of neurodegenerative diseases. Their results also showed activation of the autophagy process with the involvement of the SIRT1/ERK1/2 pathway in neuron survival regulation [98]. Moreover, SQSTM/p62 is a poly-ubiquitin-binding protein that is degraded via autophagy [99]. Degradation of p62 is a marker of autophagy activity because, after binding to microtubule-associated protein 1 A/1B-light chain 3 (LC3), it is selectively degraded via autophagy [100]. In our experiment, the M-CFS with *H. erinaceus* alone resulted in only a slight decrease in SQSTM/p62 protein expression, but after the use of the AhR antagonist, the expression significantly increased. This may indicate significant activation of the autophagy process after the blocking of AhR. This is also reflected in our results for Beclin 1 after the treatment with M-CFS with *H. erinaceus.* However, the M-CFS with *G. lucidum* alone and with CAY10464 caused a significant increase in the SQSTM/p62 expression, which may indicate an important role of AhR to ensure the appropriate metabolism of metabolites from *G. lucidum*. It can therefore be assumed that *G. lucidum* with the AhR antagonist caused the intensification of the autophagy process due to the lack of AhR. This is opposite to the results reported by Ren and coauthors [101], who reported that *G. lucidum* influenced autophagy induction through an increase in the expression of p62 and LC3 II in male C57BL6/J mice in vivo and the microglial cell line (BV-2) in vitro [101]. Interestingly, recent studies on glioblastoma cells show that AhR enhances autophagy under the influence of reduced tryptophan levels. This process provokes cells to replenish the deficiency of this amino acid, and an increase in both the number and activity of AhR can be observed [102].

## Conclusion

Our study showed that 5% M-CFS containing *H. erinaceus* or *G. lucidum* does not negatively affect the cellular metabolism and viability of the HT-22 cell line. We confirmed the antioxidant, proliferative, and survival properties of both extracts used in this experiment. Taken together, our results do not provide direct evidence for modulation of the AhR receptor by M-CFS from *H. erinaceus* and *G. lucidum*, although definitive confirmation of this mechanism will require further investigation. Our confirmation of the beneficial health-promoting effects of the tested M-CFS may justify the use of both mushrooms as a supplement to the daily diet and may contribute to the development of new food-related strategies.

## Supplementary Information

Below is the link to the electronic supplementary material.

Supplementary Material 1

## Data Availability

No datasets were generated or analysed during the current study.

## Abbreviations

Ac-DEVD-pNA: Caspase-3 colorimetric substrate
AhR: Aryl hydrocarbon receptor
ARNT: AhR nuclear translocator
BBB: Blood-brain barrier
BDNF: Brain-derived neurotrophic factor
BGM: Brain-gut-microbiome
BSA: Bovine serum albumin
BV-2: Microglial cell line
CAY10464: 1,3-dichloro-5-[(1E)-2-(4-methoxyphenyl)ethenyl]-benzene
CHAPS: 3-[(3-cholamidopropyl)dimethylammonio]-1-propanesulfonate hydrate
CHOP: C/EBP homologous protein
CNS: Central nervous system
COX-2: Cyclooxygenase-2
DMEM: Dulbecco’s Modified Eagle Medium
DRE: Dioxin-responsive element
DTT: DL-dithiothreitol
EDTA: Ethylenediaminetetraacetic acid
ERK1/2: Extracellular signal-regulated kinase-1/2
FBS: Fetal bovine serum
GSH: Glutathione
HPLC-ESI-QTOF-MS/MS: High-performance liquid chromatography–electrospray ionization–quadrupole time-of-flight–tandem mass spectrometry
HPMEC: Human fetal pulmonary microvascular endothelial cells
HSP90: Heat shock protein 90
HT-22: Mouse hippocampal cell line
IL: Interleukin
iNOS: nducible nitric oxide synthase
INT: Iodonitrotetrazolium salt
LDH: Lactate dehydrogenase
MAPK: Mitogen-activated protein kinases
M-CFS: Microbiome cell-free supernatant
MPMS: 1-methoxy-5-methylphenazinium methyl sulfate
NaCl: Sodium chloride
NAD: β-nicotinamide adenine dinucleotide
NFTs: Neurofibrillary tangles
NGF: Nerve growth factor
NO: Nitric oxide
NIH-3T3: Mouse fibroblast cell line
PBS: Phosphate-buffer saline
PCNA: Proliferating cell nuclear antigen
PI: Propidium iodide
RAW 264.7: Murine macrophage cell line
ROS: Reactive oxygen species
SOD1: Superoxide dismutase-1
SRC: Proto-oncogene tyrosine- kinase
TEMED: N,N,N′,N′-tetramethyl ethylenediamine
TNF-α: Tumor necrosis factor alpha
SHIME®: Simulator of Human Microbial Intestinal Ecosystem
