# Supplementary material for: Impact of Hericium erinaceus and Ganoderma lucidum metabolites on AhR activation in neuronal HT-22 cells
Source: Pharmacol Rep. 2025 Aug 14;77(6):1557–72. doi: 10.1007/s43440-025-00767-w (PMC12647283; doi:10.1007/s43440-025-00767-w)
Supplement: Supplementary file 1 — Supplementary Material 1 [file 43440_2025_767_MOESM1_ESM.docx]

**MEMBRANE 1**

**
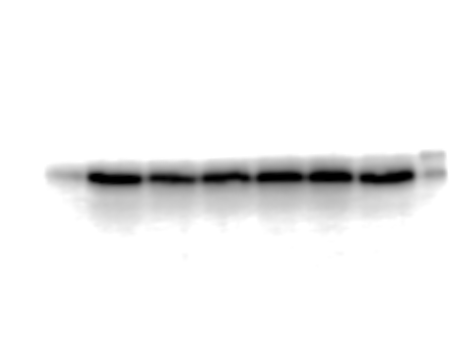
**

**SQSTM/p62 – MEMBRANE 1**

**STRIPPING**


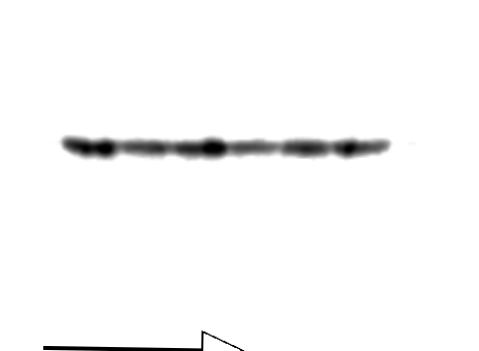

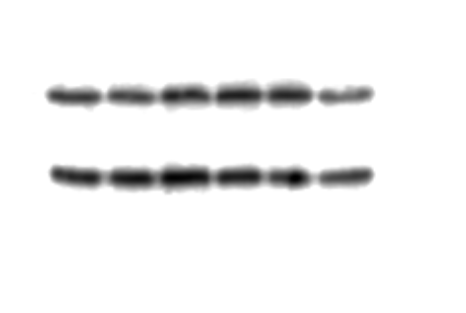


**PCNA - MEMBRANE 1**

25 kDa

48 kDa

35 kDa

20 kDa

1

2

3

4

5

6

**β-actin - MEMBRANE 1**

**STRIPPING**

**MEMBRANE 2**


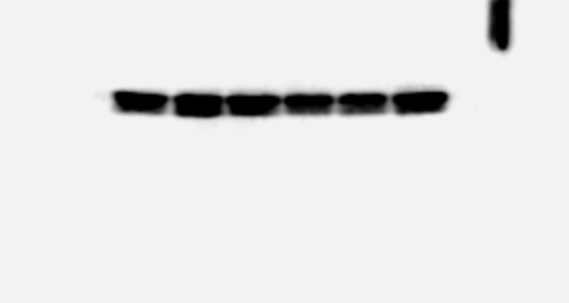


**SRC - MEMBRANE 2**


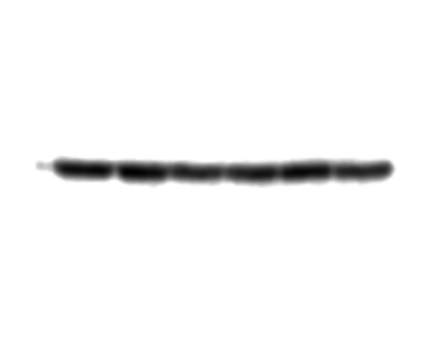


**STRIPPING**

**β-actin - MEMBRANE 2**

**STRIPPING**


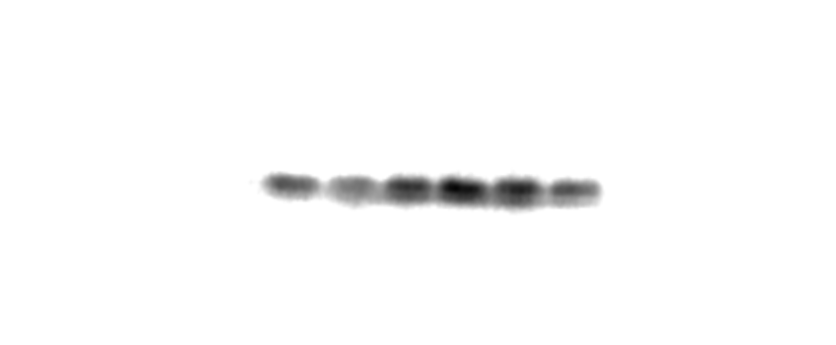


**SOD1 - MEMBRANE 2**

**MEMBRANE 3**


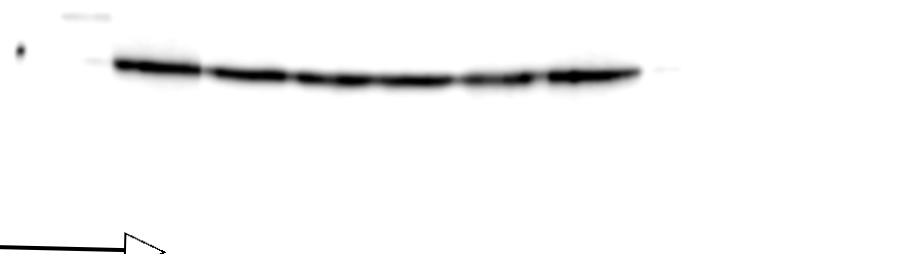

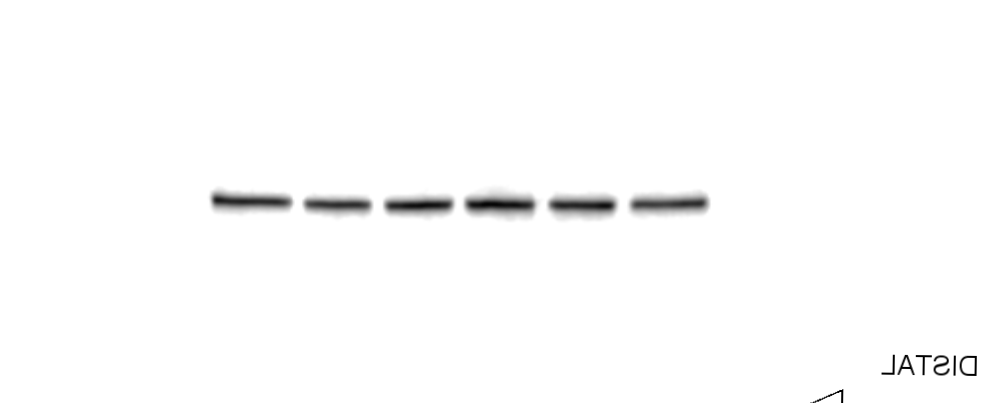


**AhR - MEMBRANE 3a**

**β-actin - MEMBRANE 3b**

**MEMBRANE 4**

**Beclin 1 - MEMBRANE 4**


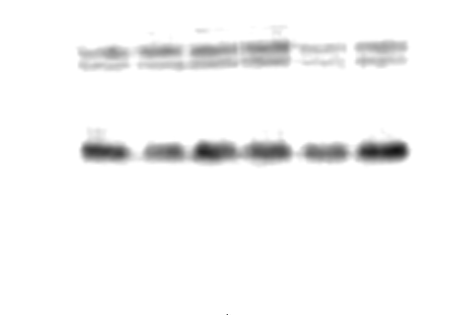


**STRIPPING**


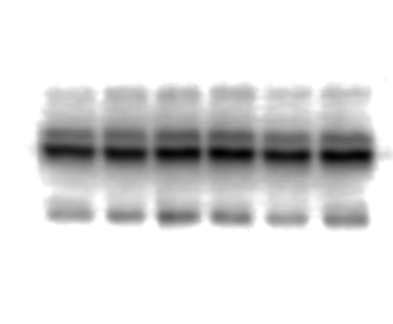


**ERK1/2 - MEMBRANE 4**

//

STRIPPING


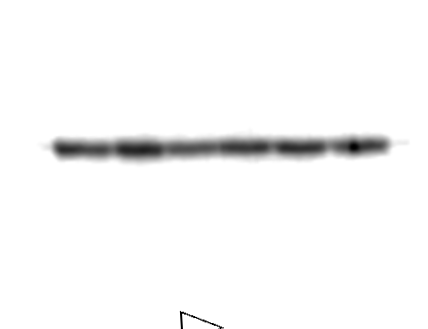


**β-actin - MEMBRANE 4**
